# Nonimmune antibody interactions of Group A *Streptococcus* M and M-like proteins

**DOI:** 10.1371/journal.ppat.1009248

**Published:** 2021-02-25

**Authors:** Jori O. Mills, Partho Ghosh

**Affiliations:** Department of Chemistry & Biochemistry, La Jolla, California, United States of America; Carnegie Mellon University, UNITED STATES

## Abstract

M and M-like proteins are major virulence factors of the widespread and potentially deadly bacterial pathogen *Streptococcus pyogenes*. These proteins confer resistance against innate and adaptive immune responses by recruiting specific human proteins to the streptococcal surface. Nonimmune recruitment of immunoglobulins G (IgG) and A (IgA) through their fragment crystallizable (Fc) domains by M and M-like proteins was described almost 40 years ago, but its impact on virulence remains unresolved. These interactions have been suggested to be consequential under immune conditions at mucosal surfaces and in secretions but not in plasma, while other evidence suggests importance in evading phagocytic killing in nonimmune blood. Recently, an indirect effect of Fc-binding through ligand-induced stabilization of an M-like protein was shown to increase virulence. Nonimmune recruitment has also been seen to contribute to tissue damage in animal models of autoimmune diseases triggered by *S*. *pyogenes* infection. The damage was treatable by targeting Fc-binding. This and other potential therapeutic applications warrant renewed attention to Fc-binding by M and M-like proteins.

## Introduction

*Streptococcus pyogenes* (Group A *Streptococcus* or Strep A) is a globally widespread gram-positive bacterial pathogen that causes a variety of diseases, ranging from mild and self-limiting (e.g., pharyngitis and impetigo) to invasive and deadly (e.g., necrotizing fasciitis and streptococcal toxic shock syndrome). In addition, autoimmune diseases (e.g., acute rheumatic fever and glomerulonephritis) can arise from Strep A infection and remain serious health threats in parts of the developing world [1–3]. Acute rheumatic fever accounts for more than half of the approximately 500,000 annual deaths due to Strep A infection [3,4]. In each of these diverse diseases, the streptococcal surface-attached M protein plays a significant role [5].

**Fig 1:**
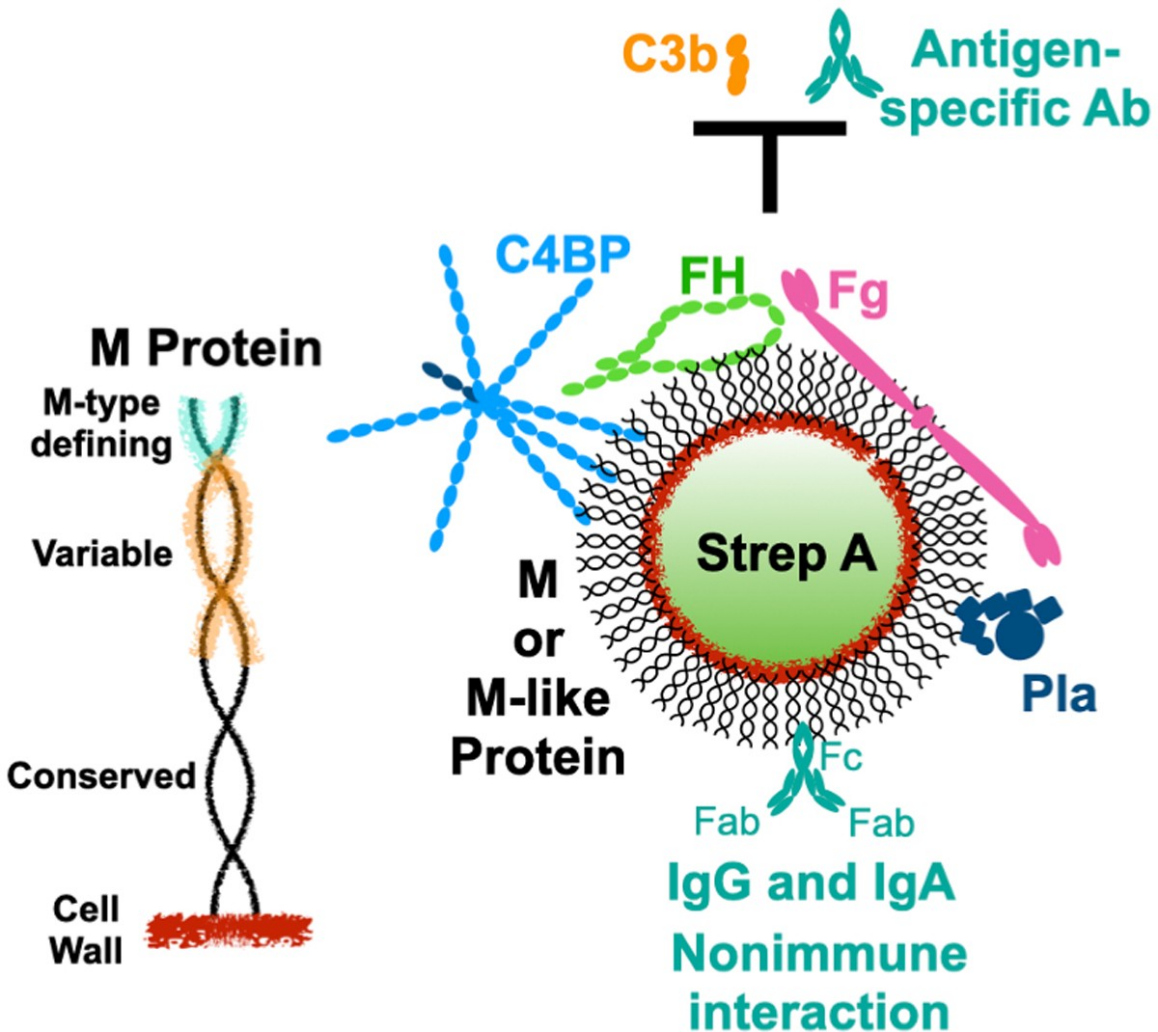
Surface recruitment of human proteins by Strep A. Left, Schematic of the M protein coiled coil attached covalently to the Strep A cell wall. The N-terminal 50 amino acids of mature M protein define its type (cyan), and the N-terminal third to half of M protein is sequence variable (orange). Right, M and M-like proteins on the Strep A surface recruit C4BP, FH, Fg, and Pla to block the deposition of opsonins, such as C3b and antigen-specific antibodies. M and M-like proteins also interact with IgG and IgA in nonimmune fashion through their Fc domains. C4BP, C4b-binding protein; Fc, fragment crystallizable; Fg, fibrinogen; FH, factor H; IgA, immunoglobulin A; IgG, immunoglobulin G; Pla, plasminogen.

M protein forms a dense fibrillar coat that extends approximately 500 Å outwards from the Strep A cell wall (Fig 1) [6]. The fibrillar appearance of M protein is due its dimeric α-helical coiled coil structure, which occurs through most of this protein’s length of approximately 330 to 440 amino acids [7–9]. The location of M protein on the bacterial surface makes it a target of immune surveillance, explaining its considerable antigenic sequence variation. The sequence of the N-terminal 50 amino acids of the mature form of M protein (i.e., after removal of the signal sequence) is hypervariable between M protein types and defines the type. More than 220 M protein types have been identified [9]. Along with the M type-defining 50 amino acids, the remaining N-terminal third to half of M protein also varies in sequence between types. In contrast, C-terminal regions are more conserved, including the “LPXTG” anchor that confers covalent attachment of M protein to the Strep A cell wall. Despite sequence variation, all M protein types appear to share a propensity for forming α-helical coiled coils [8,10–14].

One of the major functions of M protein is to provide resistance against elimination by the innate and adaptive immune systems. Resistance requires the recruitment of specific human proteins to the streptococcal surface that thwart the deposition of opsonins, such as the complement protein C3b and Strep A-specific antibodies (Fig 1). This enables Strep A to evade uptake and consequent killing by professional phagocytes. The recruited human proteins include only a handful. There are two whose mechanisms of action and structural interactions with M protein have been well studied, fibrinogen (Fg, approximately 340 kDa) [15–18] and C4b-binding protein (C4BP, approximately 570 kDa) [14,19,20], and a third which is less well studied but whose role in virulence was recently clarified through the use of a transgenic mouse model of Strep A infection, factor H (FH, approximately 150 kDa) [21]. C4BP and FH are both down-regulators of complement activation and interact with other complement proteins to decrease levels of C3b as a means to protect self-tissues from complement damage. They are co-opted by M protein to do likewise for the streptococcal surface. C4BP and FH also sterically compete with opsonizing antibodies that target their binding regions on M protein [22]. Fg is a blood clotting protein whose 450 Å-long, extended shape [23] appears to be repurposed by Strep A as a steric shield to block the deposition of complement components [17]. A fourth recruited protein also comes from the blood clotting system—plasminogen (Pla), which can be recruited directly by a specific class of M proteins, called plasminogen-binding M-like (PAM) proteins [24–26], or indirectly through streptococcal surface-bound Fg. Plasminogen is converted to the active protease plasmin by Strep A streptokinase, and plasmin has been shown to proteolyze C3b, leading to decreased opsonization by C3b and phagocytic uptake by neutrophils [27]. Surface-localized plasmin also promotes the transition of a localized streptococcal infection to an invasive one [28,29]. A fifth interaction of M protein was recognized about 40 years ago [30–34], but its role in virulence has remained unresolved. This is the nonimmune interaction of M protein with immunoglobulins G (IgG) and A (IgA) through their Fc (fragment crystallizable) domains (Fig 1).

### Fc-binding motifs

**Fig 2:**
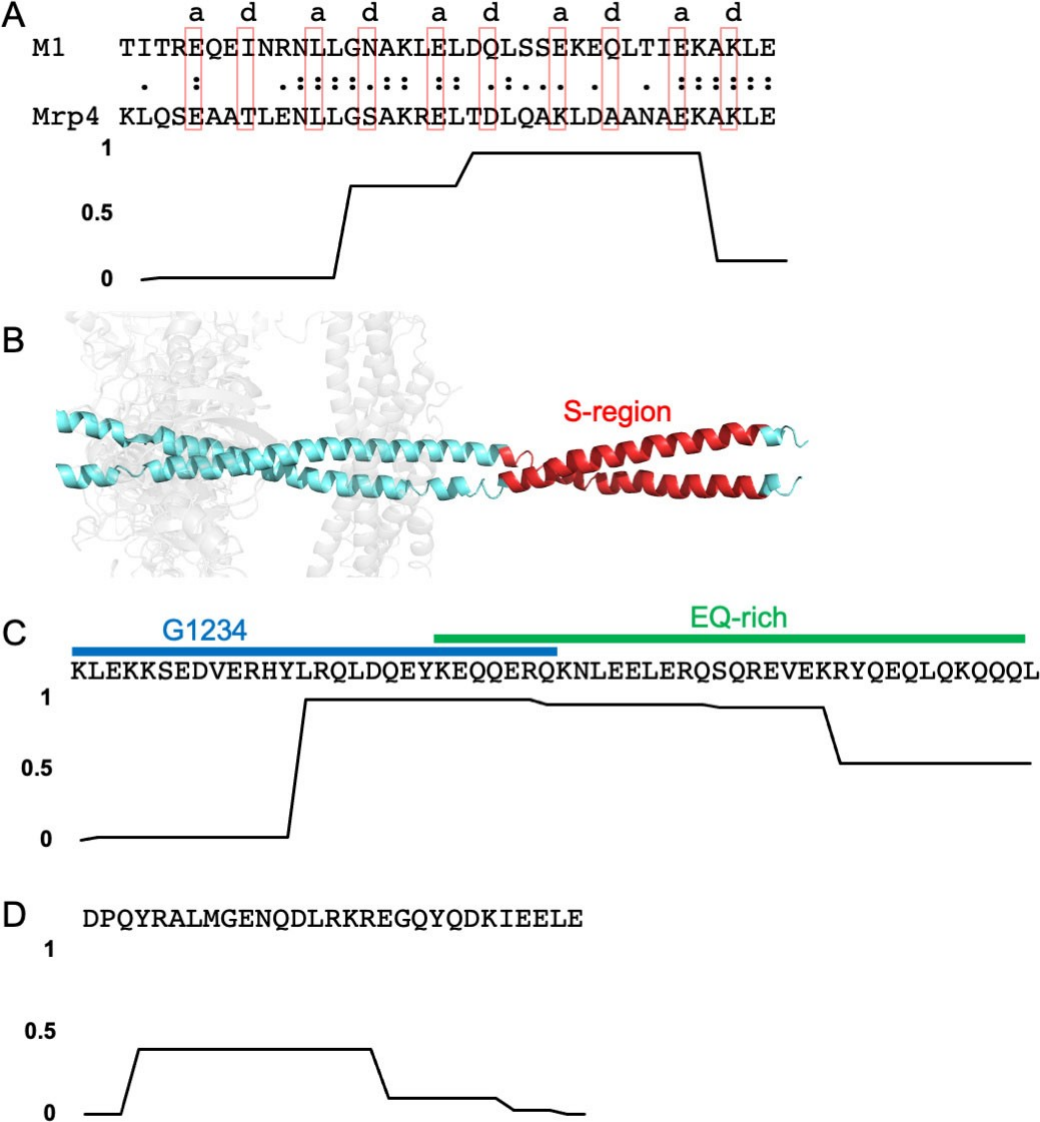
Fc-binding motifs. **(A)** S-region binding motif for Fcγ-binding. Top, the sequence of the M1 protein S-region is aligned with the S-region motif in Mrp4. Amino acids that occupy *a* or *d* positions of the coiled coil heptad repeat in the structure of the M1 S-region are denoted above the sequence and enclosed in red boxes. Bottom, coiled-coil propensity of the M1 protein S-region. (**B)** Structure of the M1 protein S-region (red) in cartoon representation. The B-repeats region of M1 protein is in cyan and fibrinogen fragment D in gray. (**C)** Top, G1234 and EQ-rich motifs for Fcγ-binding from M2 protein. Bottom, coiled-coil propensity of the sequence at top. (**D)** Top, Fcα-binding motif from M4 protein. Bottom, coiled-coil propensity of the sequence at top.

A number of M protein types have been shown to bind human IgG-Fc (Fcγ), IgA-Fc (Fcα), or both. IgG is primarily found in plasma but can also be found on mucosal surfaces, while IgA is the primary antibody class found on mucosal surfaces [35]. There are 3 Fc-binding motifs which are easily discernable in primary sequence, with two of these responsible for binding Fcγ. One of the Fcγ-binding motifs is found in the S-region of M1 protein (Fig 2A and 2B) [36] and in a few other M proteins [37], including M55 and M64 proteins [38,39]. The 38-amino acid S-region occurs near the middle of M1 protein, at the very end of its variable region. The M1 protein S-region has been seen to form a dimeric α-helical coiled coil as part of a larger M1 protein fragment that is bound to a fragment of Fg (Fig 2B) [18]. The coiled coil, however, has some irregularities in its structure, with a number of the core *a* and *d* positions of the heptad repeat being occupied by destabilizing amino acids (e.g., Glu at *a* positions) [40,41]. Alpha-helical coiled coils have a heptad repeat pattern in their primary sequence in which the *a* and *d* positions form the core of the structure and are typically occupied by Val and Leu, respectively, in dimeric, parallel coiled coils. The ends of the S-region sequence have low propensities for coiled-coil formation (Fig 2A), and the observed coiled-coil structure of the S-region is likely due to Fg providing ligand-induced stabilization, a mechanism described below.

The second Fcγ-binding motif is unrelated to the S-region motif and occurs in a larger number of M proteins. This motif appears to be bipartite. The first part is a 28-amino acid sequence called G1234 and is followed by or overlaps with a 35-amino acid sequence called the EQ-rich region; the composition of this second part is >50% Glu or Gln (Fig 2C) [42,43]. This bipartite sequence has a high propensity for forming a coiled coil near the end of G1234 and beginning of the EQ-rich motifs. The G1234 and EQ-rich motifs are usually found in tandem, with some exceptions. For example, M49 protein contains only the EQ-rich motif. Additionally, M2 protein is unusual in containing all 3 Fcγ-binding motifs—G1234, EQ-rich, and S-region.

In contrast to there being 2 Fcγ-binding motifs, only 1 Fcα-binding motif has been identified. This is a 29-amino acid sequence that has a low propensity of forming a coiled coil (Fig 2D) [33,34]. A number of M proteins, such as M22, contain both Fcγ- and Fcα-binding motifs. In M22 protein, these sites are located closely enough that steric hindrance prevents simultaneous binding of IgG and IgA [44].

### M-like proteins

These same Fcγ- and Fcα-binding motifs also occur in M-like proteins, which are encoded proximally to *emm* in a large number of Strep A strains. Namely, these are Enn proteins [33,42] and M-related proteins (Mrp) [45], which are encoded immediately downstream or upstream, respectively, of *emm* in the *mga* regulon [46,47]. A recent survey of a collection of 1,688 globally distributed Strep A isolates showed that approximately 85% encode both Enn protein and Mrp [48]. Enn proteins are sequence variable but to a lesser extent than M proteins [48,49], and Mrps are even less sequence variable [50]. For example, in the aforementioned survey of Strep A isolates, the pairwise identity of M proteins, Enn proteins, and Mrps was 45%, 69%, and 83%, respectively [48]. Mrps have the S-region motif for Fcγ-binding (Fig 2A), which appears to be conserved among Mrps encoded by Strep A strains of differing M types [45,51–53]. An additional M-like protein is responsible for nonimmune interaction with antibodies, Protein H (PrtH), which appears to be found exclusively in M1 Strep A strains and has the bipartite G1234 and EQ-rich motif for Fcγ-binding [43] (Fig 2C). PrtH is encoded immediately downstream of *emm1* [54] in approximately 30% of M1 Strep A strains [55]. These M-like proteins appear to have arisen through gene duplication events [56–58], and their sequences have heptad repeat patterns diagnostic of α-helical coiled-coil structure [59]. Alpha-helical character in Mrp4 [13] and PrtH [12] has been confirmed by circular dichroism analysis, and Mrp4 has been shown to form a more stable α-helical coiled coil than a typical M protein, M4 [13].

### High affinity interaction with Fc

**Fig 3:**
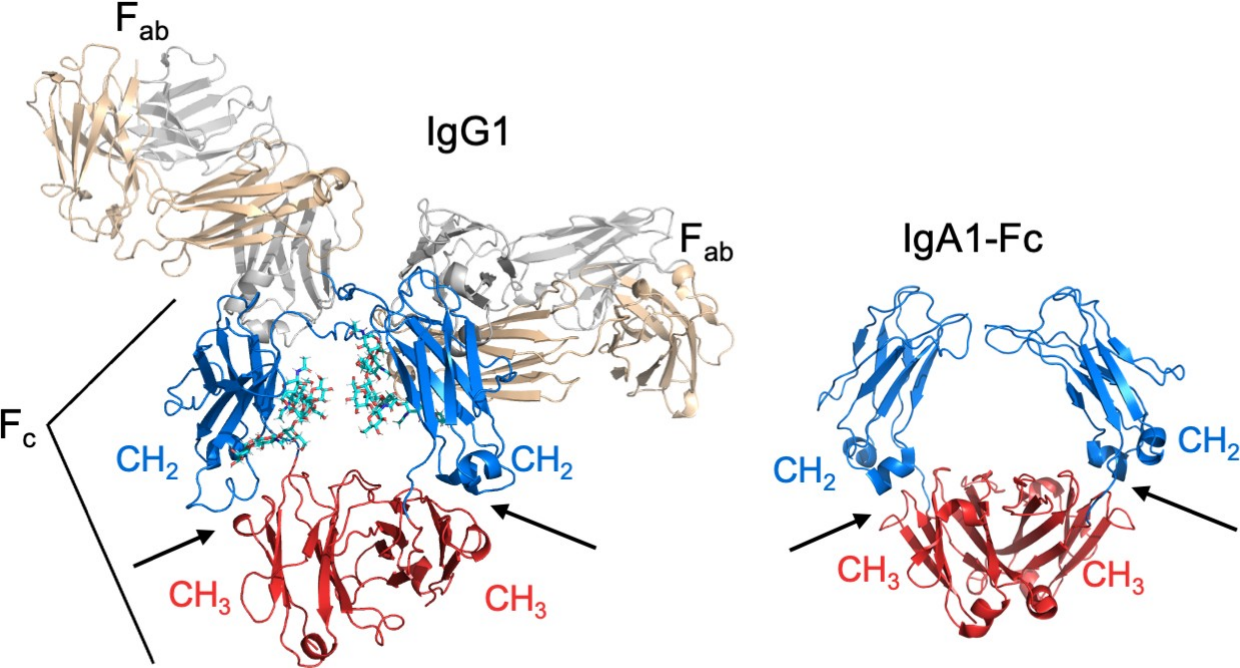
CH_2_–CH_3_ interface. Structure of IgG1 (PDB 1IGY), left, and IgA-Fc1 (PDB 2QEJ), right, in cartoon representation. The interfaces between the CH_2_ (blue) and CH_3_ (red) domains are indicated by arrows.

The affinity of M and M-like proteins for IgG-Fc via the bipartite G1234 and EQ-rich motifs has been measured to be quite high. The G1234- and EQ-rich motif-containing M22 protein has a dissociation constant (K_D_) of approximately 1.3 nM for polyclonal IgG [44], and similar K_D_’s were found for the interaction of the G1234- and EQ-rich motif-containing PrtH with both Fcγ (1.6 nM) and intact polyclonal IgG (0.4 to 0.6 nM) [54,60]. M22 protein and PrtH have been shown to bind all 4 IgG subclasses (i.e., IgG1, IgG2, IgG3, IgG4, which differ in the hinge and CH_2_ and CH_3_ domains) (Fig 3) [44,61], with PrtH having a slight preference for IgG3 [60]. It has been suggested that the G1234 motif directs binding to all 4 IgG subclasses while the EQ-region directs binding to only IgG3, and that these 2 motifs can function independently of each other [43]. However, further study of these issues is warranted, as these conclusions were not based on examination of purified proteins but rather crude extracts or inclusion bodies containing fusion proteins that were not directly refolded [42,43,51].

In contrast, the S-region in purified form has been shown to be sufficient for Fcγ-binding; this involved a truncation fragment of Mrp4 [45]. The affinity of the M1 protein S-region for IgG has been determined, but the results are discordant. M1 protein was found in several reports to bind weakly to polyclonal IgG [11,36], with a K_D_ of only 0.3 to 10 μM, whereas another study reported a 5-nM dissociation constant [37]. The reason for this discrepancy is not clear. In addition, M1 protein appears to bind all 4 IgG subclasses [36], while Mrp4 shows a preference for IgG1>IgG4>IgG2>IgG3 [45]. Thus, further work needs to be done to determine the affinity and subclass preference of the S-region motif.

Like the S-region of Mrp4, the Fcα-binding motif in purified form has been shown to be sufficient for Fcα-binding; this involved a synthetic peptide corresponding to M22 protein [62]. The Fcα-binding motif has a strong affinity for polyclonal IgA, exhibiting K_D_’s in the sub- to low nanomolar range [37,44,63,64]. A measurement with isolated Fcα has not been reported. Both IgA1 and IgA2 subclasses are bound by M22 protein, along with (secretory) sIgA1 and sIgA2 [63]; the latter are dimeric forms of IgA complexed with the proteins J-chain and secretory component. While M22 protein bound monomeric serum IgA and dimeric sIgA with similar affinity [63], M4 protein had a 10-fold lower affinity for sIgA as compared to serum IgA and similarly a 10-fold lower affinity for IgA complexed with α_1_-microglobulin [64].

It is worth noting that the Fc-affinity of M and M-like proteins on the Strep A surface may be modulated by other molecules. For example, in the case of an M1 Strep A strain that expresses PrtH, binding by the bacterium to a monoclonal IgG via the Fcγ region was almost entirely attributable to PrtH; M1 protein contributed almost nothing [65]. However, in the same study, binding by the streptococcal surface-attached PrtH to IgG in saliva via the Fcγ region was enhanced by M1 protein [65]. The mechanism behind this enhancement is unknown. Likewise, as noted above, PrtH as a purified protein shows preference for IgG3 [60], but on the streptococcal surface binds little IgG3 and instead prefers IgG1 [65].

### Interference with antibody effector function

The target of M and M-like proteins for both IgG-Fc and IgA-Fc is the interface between the CH_2_ and CH_3_ domains (Fig 3) [36,52,66,67]. Significantly, the CH_2_–CH_3_ interface is critical for interactions of IgG and IgA with immune effector molecules. Evidence indicates that this antibody region is especially suited for protein–protein interactions [68]. The binding of IgG and IgA at the CH_2_–CH_3_ interface by M and M-like proteins competes directly with the interaction of these antibodies with host immune effector molecules and thereby blocks these effector functions.

For IgG1 and IgG3 subclasses, these effector functions include interaction with complement C1q [69,70]. C1q binds the IgG1 and IgG3 subclasses at the Cγ_2_–Cγ_3_ interface (Fig 3) and, when clustered due to binding of antigen-clustered IgGs, activates the classical complement pathway. The Cγ_2_–Cγ_3_ interface is also critical for interaction of IgG1 and IgG3 subclasses with Fcγ receptors, such as the high-affinity FcγRI (CD64). Fcγ receptors are found on monocytes, macrophages, neutrophils, and other immune cells, and their activation by antigen-clustered IgG1 or IgG3 can lead to phagocytic uptake, production of reactive oxygen species, along with the release of proinflammatory mediators among other effects.

Unlike IgG, IgA does not interact with C1q or the complement system and instead functions by binding the receptor FcαRI (CD89) through its Cα_2_–Cα_3_ interface (Fig 3) [71]. FcαRI is expressed on neutrophils, monocytes, macrophages, eosinophils, dendritic cells, and Kupffer cells, and its activation by antigen-clustered IgA can result in phagocytic uptake and proinflammatory responses, such as the production of reactive oxygen species and release of proinflammatory cytokines.

Thus, nonimmune binding by M and M-like proteins of anti-Strep A antibodies prevents such antibodies from activating complement, interacting with host phagocyte receptors, or both.

### Immune environment

When during Strep A infection does nonimmune interaction by M or M-like proteins matter? Certainly, evidence suggests that nonimmune interactions provide a steric shield against the deposition of complement [22] and antibodies that are specific to the Fc-binding sites of M and M-like proteins [20,67]. But what about antibodies that are specific for other regions of M and M-like proteins, or other streptococcal surface antigens? Can nonimmune antibody interactions eliminate or at least greatly reduce anti-Strep A antibody interactions that lead to opsonization?

A study suggests that the answer is no [65]. This study made clever use of the *S*. *pyogenes* IgG-cleaving protease IdeS to cleave Fab from Fc on IgGs bound to the streptococcal surface; the antibody fragment remaining on the surface was then identified using specific reagents. The source of IgG was either IVIG (pooled from >3,500 healthy volunteers), or pooled plasma or saliva from 5 individuals. In each of these samples, substantially high levels of anti-Strep A IgGs were found, as assessed by their specificity being Fab- (fragment antigen-binding) rather than Fc-directed [65]. This study used an M1 Strep A strain that also expressed PrtH, and these anti-Strep A IgGs were found to be mostly directed against streptococcal surface antigens other than M1 protein and PrtH. This study found that at high concentrations of anti-Strep A IgGs, as in plasma, Fcγ-directed binding was overwhelmed by Fab-directed binding. However, at low concentrations of anti-Strep A IgGs, as in saliva (measured to be approximately 10^4^ lower in concentration than in plasma), Fcγ-directed binding due to PrtH was dominant over Fab-directed binding. This study suggests that nonimmune IgG interaction of Strep A is insignificant at high concentrations of anti-Strep A IgG, as in plasma, but significant at low concentrations of anti-Strep A IgG, as on mucosal surfaces and secretions, which are natural habitats for Strep A.

This study further showed that, at the low IgG concentration of saliva, the loss of PrtH affected intracellular survival of Strep A in neutrophils rather than phagocytic uptake. In the absence of PrtH, no difference in uptake was seen but intracellular survival decreased by about half [65]. This effect was presumably due to the Fcγ-binding capacity of PrtH but was not isolated to this interaction. This effect was also consistent with other studies that showed that PrtH (and M1 protein) increase the intracellular survival of Strep A in neutrophils by blocking the fusion of azurophilic granules with phagosomes [72,73]. The precise mechanism for this block in vesicle fusion brought about by PrtH and M1 protein is unknown, and whether survival is dependent on nonimmune interaction with IgG is likewise unknown.

A similar study has not been carried out for IgA. While nonimmune interaction of M4 protein with IgA has been shown to block the respiratory burst in neutrophils in vitro [67], further study is required to discern the milieu in which nonimmune IgA interaction impacts virulence. Such studies may also be able to examine the relative contributions of nonimmune IgG and IgA interaction, for example, using an M22 strain since M22 protein binds both IgG and IgA and these 2 functions appear to be dissociable [20].

### Nonimmune environment

Several studies have shown Fc-directed interaction enhances survival of Strep A in nonimmune human blood. In one study, short internal deletions were made in M22 protein to eliminate either Fcα-binding or C4BP-binding, while leaving Fcγ-binding intact [20]. Based on these deletions, the combined effects of Fcα- and C4BP-binding in resisting killing in nonimmune human blood were found to be cooperative. That is, loss of both interactions led to a strain with approximately 20-fold less resistance to killing, equivalent to the resistance of a strain lacking M22 protein entirely, but loss of the individual interactions was nonadditive, each resulting in only approximately 2-fold less resistance to killing. While C4BP-binding was shown to down-regulate complement activation by the classical pathway, Fcα-binding had no effect on complement activation. The mechanism of resistance afforded by Fcα-binding was unclear.

A similar effect on resistance to killing in nonimmune human blood was observed for the interaction of Mrp4 with IgG-Fc [45]. In an M4 Strep A strain that expressed Mrp4, M4 protein, and Enn4 protein, it was found that Mrp4 was the major Fcγ binder [45]. An internal deletion in Mrp4 that severely decreased binding to Fcγ but left binding to Fg at nearly wild-type level resulted in an approximately 5-fold reduction in growth in nonimmune human blood as compared to the wild-type M4 Strep A strain [45]. However, once again, the mechanism of protection against phagocytic killing conferred by Fc-binding was unclear. It must be mentioned that, in a separate study using a different M4 Strep A strain, no effect of Mrp4 on survival in human blood was observed [51], but that strain also grew quite poorly in blood and may have been attenuated.

Resistance against killing in nonimmune blood conferred by Fc-binding has been suggested to be due to action against anti-Strep A natural antibodies [20]. Natural antibodies are present before the introduction of an antigen and are relatively nonspecific and polyreactive [74]. Evidence for the incidence of natural anti-Strep A IgA exists [75], but whether such antibodies were present in the blood samples used in the aforementioned experiments and disabled due to nonimmune recruitment was not determined. Further study is required to definitively determine how Fc-binding aids in resistance to killing in nonimmune blood.

### Ligand-induced stabilization through Fcγ-binding

An indirect role for Fcγ-binding separate from its effector function has recently been described [55,60]. PrtH was observed to bind C4BP poorly at 37°C, but upon the addition of IgG, bound C4BP with greatly enhanced affinity [55]. This IgG could be in the form of nonimmune monoclonal IgG, polyclonal IgG, or purified Fcγ, and enhancement was seen whether PrtH was soluble or attached to the streptococcal surface. This enhanced affinity enabled a PrtH-expressing Strep A strain, given both C4BP and IgG, to survive approximately 2.5-fold better against killing by neutrophils as compared to the same strain given C4BP alone [55]. Consistent with these findings, the PrtH-expressing Strep A strain was found to be considerably more virulent when Fcγ or a nonimmune monoclonal IgG was coadministered in a systemic murine model of infection [55]. This was in comparison to mock coadministration, and this model utilized humanized transgenic C4BP mice to provide C4BP, as murine C4BP does not bind M or M-like proteins [76].

**Fig 4:**
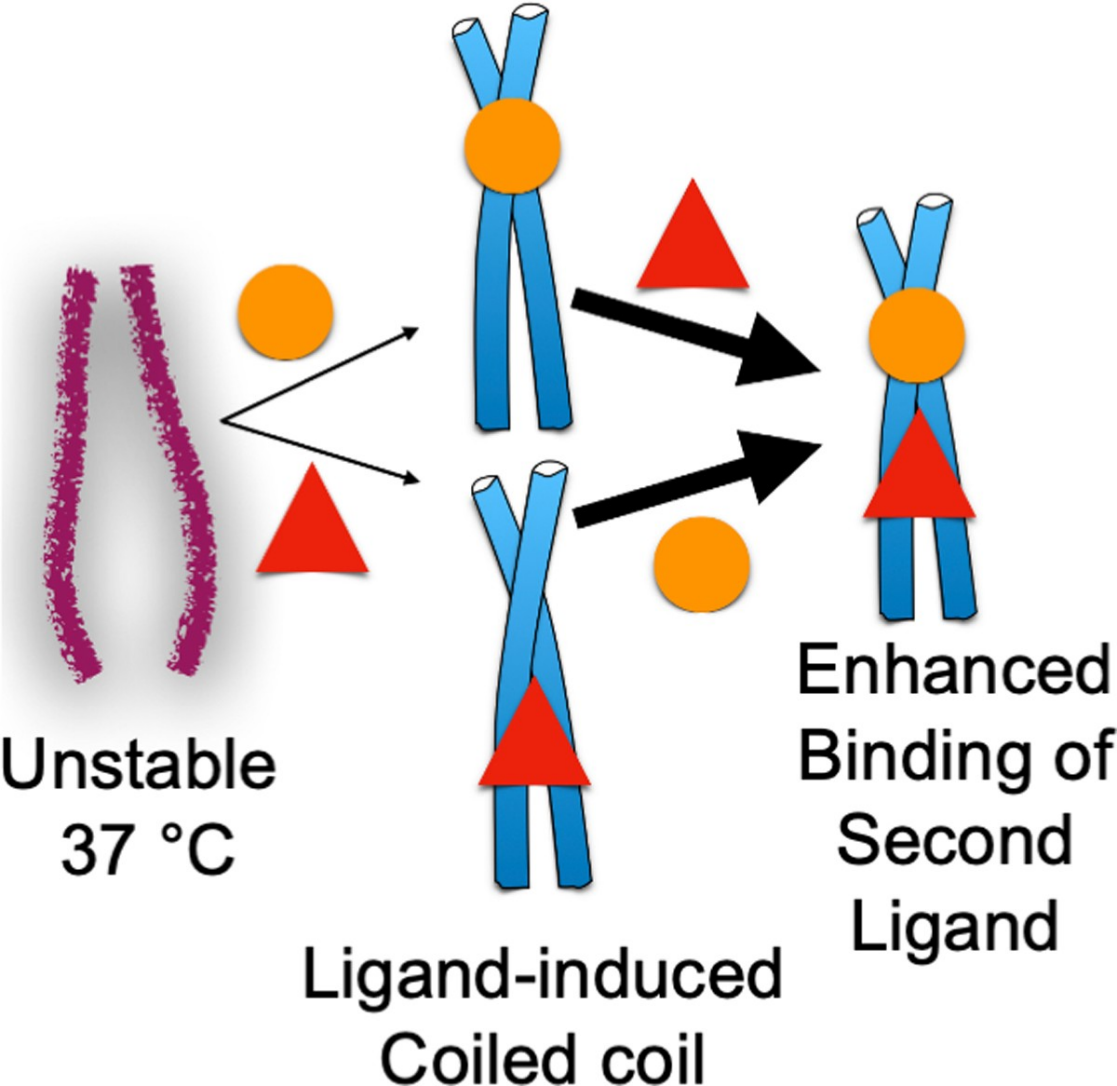
Ligand-induced stabilization. M and M-like protein coiled coils are often unstable at 37°C but can be stabilized by the low-affinity binding (denoted by thin arrows) of a ligand (circle or triangle). Formation of coiled-coil structure promotes the high-affinity interaction (denoted by thick arrows) of a second ligand.

The enhancement in C4BP affinity by Fcγ binding to PrtH appears to be due to ligand-induced stabilization (Fig 4). PrtH along with several M proteins have been shown to be unstable at 37°C. At this temperature, these molecules dissociate from a dimeric α-helical coiled coil state to a monomeric state that has diminished α-helical character [10,12,13]. Consistent with the loss of structure at 37°C, M and M-like proteins have been shown to bind poorly at 37°C to IgG and IgA and other ligands [10,11,13]. However, at lower temperatures (4 to 26°C), which stabilize coiled coil structure, binding occurs well [10,11,13].

Notably, the instability of the M and M-like protein coiled coil has been shown to be counteracted by the binding of a ligand (Fig 4). For example, the binding of IgG to PrtH was found to stabilize the PrtH coiled coil [12], as was the binding of albumin, which binds to the conserved C-repeats region of M and M-like proteins [77]. The combined binding of IgG and albumin had a synergistic effect in stabilizing the coiled-coil structure of PrtH [12]. Consistent with this synergistic effect, stabilization of the M1 protein coiled coil by the binding of 1 ligand was seen to enhance the binding of a second ligand (Fig 4). Namely, while M1 protein bound IgG and Fg poorly at 37°C, if one of the ligands were prebound to M1 protein using a lower temperature, then the other ligand bound well at 37°C [11]. This effect was seen to be reciprocal between IgG and Fg. The binding sites for IgG-Fc and Fg in M1 protein are proximally located (Fig 2B) [18], and the effect of 1 binding event on the other appeared to be localized as no large global structural stabilization of M1 protein was evident due to IgG binding [12]. In this mode of reciprocal enhancement, the first binding event is low-affinity as binding energy needs to be expended to cause the coiled coil to form, whereas the second binding event is high-affinity as the coiled-coil structure is extant (Fig 4). A coiled-coil structure has been shown to be required for interaction of M protein with C4BP and Fg [14,18] and appears to be the case as well for Fc domains. This ligand-induced stabilization appears to explain the enhancement of C4BP-binding to PrtH by Fcγ-binding, although reciprocity between Fcγ- and C4BP-binding was not explored [55,60].

It is important to note that not all coiled coils are unstable and not all ligands promote stabilization. The Mrp4 coiled coil has been noted to be quite stable, and its binding to Fg does not decrease at 37°C [13]. In contrast to the reciprocal stabilization provided by IgG and Fg to M1 protein [11], IgG and albumin had no such effect on each other’s binding to M1 protein [11]. This was also the case for M22 protein and its interactions with IgA and C4BP. Despite the binding sites in M22 protein for IgA-Fc and C4BP being proximal to one another, deletion of the Fcα-binding site did not markedly attenuate binding to C4BP and vice versa [20]. Further experiments are required to understand why certain pairs of ligands induce stabilization whereas others do not.

### Autoimmune diseases

Nonimmune binding of IgG appears to have a profound role on autoimmune diseases that follow Strep A infection, such as acute rheumatic fever and glomerulonephritis. It appears that IgG bound via their Fc domains to the streptococcal surface elicit anti-IgG antibodies and the formation of multiprotein immune complexes, leading to tissue deposition of IgG and C3 [78]. In support of this, when M1 or M22 Strep A strains, which both bind Fcγ, were injected intravenously into rabbits, myocardial damage, as occurs in acute rheumatic fever, was seen [78]. In contrast, myocardial damage was absent when a Strep A strain that does not bind Fcγ was injected [78]. Rabbit IgG-Fc is bound by M and M-like proteins, albeit less well than human IgG-Fc [36,45]. Similarly, an M22 strain deleted of its Fcγ-binding proteins (i.e., M22 protein and Mrp) did not elicit myocardial damage [78]. Degenerative damage of the glomerulus, as is seen in glomerulonephritis, was evoked in rabbits through intravenous introduction of Strep A strains that bind Fcγ but not by those that do not bind Fcγ [79]. Notably, it was found that purified M22 protein and Mrp were sufficient to cause glomerular damage and that the glomerular damage caused by an Fcγ-binding M1 Strep A strain could be blocked by prior administration of human or rabbit Fcγ [80].

### Note added in proof

A paper analyzing interactions between Fcγ and M1 protein (Khakzad et al., PLoS Comput Biol. 2021;17(1):e1008169. Epub 2021/01/08. doi: 10.1371/journal.pcbi.1008169) was published while this review was in proof.

## Conclusions

Nonimmune binding of IgG, IgA, or both is conserved in a wide variety of M protein types, Mrps, and PrtH. Mrps are encoded in almost 90% of Strep A isolates as surveyed from a globally representative collection [48], and PrtH is encoded in 30% of M1 Strep A strains, with M1 being the most prevalent M type in industrialized nations and the most prevalent cause of invasive disease in the United States [81,82]. It is therefore more likely than not that a Strep A strain interacts in nonimmune fashion with IgG, IgA, or both. How such interactions contribute to virulence remains unresolved. A problem in answering this question has been the immune status of human blood used for experiments. While more thorough characterization of human blood is warranted in future experiments (e.g., for the presence of natural anti-Strep A antibodies), a rabbit model may offer some benefits. In contrast to murine Fc, which does not bind M and M-like proteins, rabbit Fc does, albeit at approximately 3- to 4-fold lower affinity than human Fc [45]. Strep A is restricted to humans, and thus nonimmune rabbits would not be expected to have high levels of anti-Strep A antibodies. It is not clear whether the weaker binding of rabbit as compared to human Fc to M and M-like proteins would provide an impediment, but the rabbit model has been useful in studying autoimmune sequelae of Strep A infection [79].

Fc-binding sites in M and M-like proteins are easily discernable in M and M-like protein primary sequences. This is unusual in that sites for other ligands, such as C4BP, factor H, and Fg, are not. Structural results indicate the conservation of a three-dimensional pattern for binding C4BP in some M proteins, one that is only weakly evident in primary sequence [5,14]. Why Fc-binding motifs are “exposed” in primary sequence is not clear, but this exposure opens up the possibility of exploiting Fc-binding motifs as antigens that may elicit a broad protective immune response against a variety of Strep A strains. Indeed, Mrps have been shown to be immunogenic and elicit bactericidal antibodies [83]. In addition, targeting Fc-binding by M protein has already been shown to have therapeutic potential in an animal model of glomerulonephritis [80]. The potential therapeutic benefit of targeting Fc-binding warrants renewed research efforts on nonimmune interactions by M and M-like proteins.
